# Maternal mortality in Egypt during the COVID-19 pandemic using record-based data from January 2020 to December 2021

**DOI:** 10.1038/s41598-025-15818-8

**Published:** 2025-08-30

**Authors:** Shaimaa Abdallah Gebili, Norhan Hassan Hekal, Neamat Hamdy Elsawy, Esraa Morshedy Beltagy, Walaa Mohamed Kandil, Ebtisam Hassanin, Hatem gamaleldin, Mahmoud M. Tolbah, Ahmed Mohamed Abady, Ramy Mohamed Ghazy

**Affiliations:** 1 Head of Clinical Research Department of Fayoum Government, Ministry of Health and Population, Cairo, Egypt; 2 Biomedical Informatics and Medical Statistics, Medical Research Institute, Alexandria University, Alexandria, Egypt; 3 Clinical pharmacy inspection, Fowa Health administration, Ministry of Health and Population, Kafr Elsheikh, Egypt; 4 Clinical Research Department, Kafer Elsheikh Health Affairs, Ministry of Health and Population, Kafr Elsheikh, Egypt; 5 Clinical Research Coordinator at Beheira Health Affairs, Ministry of Health and Population, Damanhour, Egypt; 6 Clinical Pathology Department, Faculty of Medicine, New Valley University, Kharga, Egypt; 7 Ministry of health and Population, Cairo, Egypt; 8 Tropical Health Department, High Institute of Public Health, Alexandria University, Alexandria, Egypt; 9 Family and Community Medicine, College of Medicine, King Khalid University, Abha, Saudi Arabia

**Keywords:** Maternal mortality, Egypt, COVID-19, Antenatal care, Maternal health, Diseases, Health care, Medical research, Risk factors

## Abstract

This study aimed to identify the characteristics and predictors of coronavirus disease (COVID-19)-related maternal deaths in 2020 and 2021, and to assess maternal mortality ratio (MMR) from 2018 to 2021 in Egypt. A record-based cross-sectional analytical study was conducted in four randomly selected governorates: Kafr El-Sheikh, El-Behira, (Lower Egypt) and Assiut, and Fayoum (Upper Egypt). Data from 541 maternal deaths were analyzed, revealing that 37.7% occurred in Assuit, 28.5% in El-Behira, 22.7% in Fayoum, and 11.1% in Kafr El-Sheik. The mean age of the studied population was 28.9 ± 6.4 years, with 39.0% having 1–2 children and 26.8% being nulliparous. Direct causes, including postpartum haemorrhage, preeclampsia, and embolism, accounted for 47.5% of deaths. As one of indirect causes of deaths, COVID-19 was diagnosed in 25.3%, it was the sole cause in 75.0% of them. Multivariable analysis identified the year 2021 (adjusted odds ratio (aOR) = 3.32; 95% CI, 1.9–5.81), residence in Lower Egypt (aOR = 5.15; 95% CI, 2.61–10.18), and hospital referral refusals (aOR = 8.72; 95% CI, 1.73–44.0) as key predictors of COVID-19-associated MM. The overall MMR increased between 2018 and 2021 with significant increases observed in Fayoum (from 39.25 to 71.65; *p* < 0.001) and Kafr El-Sheikh (from 36.44 to 56.6; *p* = 0.032). Yearly comparisons revealed significant inter-governorate differences in all years except 2021 (*p* = 0.15), with Assuit maintaining the highest MMR. Although the national MMR increased from 44.1 (pre-COVID-19) to 55.9 (post-COVID-19), this change was not statistically significant (*p* = 0.236). The findings highlight an alarming rise in maternal deaths and underscore the need for targeted interventions to address the direct causes of MM and improve healthcare access during crises like the COVID-19 pandemic.

## Introduction

Maternal mortality (MM) is described by the tenth revision of the International Classification of Diseases (ICD-10) as the death of a woman caused by complications related to pregnancy or medical interventions during or shortly after pregnancy, typically within 42 days of delivery. This excludes deaths due to accidental or incidental reasons. MM is a critical indicator of a country or region’s overall health status, reflecting the effectiveness, accessibility, and quality of its healthcare system^1^.

Goal 3.1 of The Sustainable Development Goals (SDGs) aims to lower the global maternal mortality ratio (MMR) to fewer than 70 maternal deaths per 100,000 live births by 2030^2,3^. MM encompasses a wide range of factors, both obstetric and non-obstetric, that collectively impact maternal health outcomes. These causes are broadly categorized into direct and indirect causes. Direct maternal deaths result from obstetric complications during pregnancy, labor, or the postpartum period, as well as from interventions, omissions, or incorrect treatment. These include conditions such as obstetric hemorrhage, hypertensive disorders, complications related to anesthesia, and adverse outcomes of cesarean section (CS)^4^. In contrast, indirect maternal deaths are linked to underlying health conditions or illnesses that emerge during pregnancy but are not directly triggered by pregnancy-related medical procedures. These conditions are exacerbated by the physiological changes associated with pregnancy and may include chronic illnesses such as cardiovascular or renal diseases^5^.

In 2020, it was estimated that around 287,000 women globally died from maternal-related causes. In the United States (U.S.), the MMR for 2021 was 32.9 per 100,000 live births, compared to a ratio of 23.8 in 2020 and 20.1 in 2019. On the other hand, Sub-Saharan Africa was the only region with the highest MMR, estimated at 545 maternal deaths per 100,000 live births, with three sub-regions having a very high MMR in 2020 (Western Africa at 754, Middle Africa at 539, and Eastern Africa at 351)^6^. In Egypt, the MMR declined significantly from 174 deaths per 100,000 live births in 1990 to 52 per 100,000 in 2013. By 2017, the MMR had further decreased to 37 per 100,000, and it reached 17 per 100,000 live births in 2020^7^.

In recent decades, studies have consistently demonstrated that newly emerging strains of influenza and coronaviruses, known for causing severe respiratory illnesses, tend to have a greater impact on pregnant women. This is partly due to the unique immunological and cardiopulmonary adaptations that occur during pregnancy^8–10^. During three major influenza pandemics in the past century (1918, 1957–1958, and 2009), pregnant women in their second or third trimester were significantly more susceptible to hospitalization or mortality compared to the general population^11^.

One of the indirect causes of maternal deaths that occurred in the world recently was the coronavirus disease 2019 (COVID-19). The COVID-19 pandemic clearly revealed significant differences in vulnerability and fragility at both the individual and health system levels, within and between countries^12,13^. As of August 2023, nearly 770 million confirmed cases and around 7 million deaths have been reported globally^14^.

Women infected with COVID-19 during pregnancy have a higher chance of being hospitalized, needing admission to intensive care units (ICUs), or mechanical ventilation compared to non-pregnant women^15^. Additionally, infection during pregnancy has been linked to a higher rate of CS^16^ and several adverse pregnancy outcomes, including preeclampsia, preterm birth, and stillbirth—particularly in cases of severe disease^17^.

A systematic review and meta-analysis reported a significant increase in maternal and fetal mortality among women infected with severe acute respiratory syndrome coronavirus 2 (SARS-CoV-2), the causative agent of COVID-19, especially in low- and middle-income countries (LMICs)^18^. In line with these findings, Allottey et al.,^2^ estimated that 970 pregnant women (0.02%) out of 179,981 with confirmed COVID-19 died from any cause across 192 studies^9^. Interestingly, however, early reports, mainly from China, suggested that SARS-CoV-2 does not exhibit the same exacerbated disease severity in pregnancy as observed in previous influenza and coronavirus outbreaks^19^. Beyond the direct effects of the virus, there is evidence that the pandemic and its effects on healthcare systems also affected the quality of life and well-being of pregnant women^20^, with increased reports of maternal depression^18^ .

This study pursued several key objectives. First, it aimed to identify the specific characteristics of maternal deaths occurring in 2020 and 2021. Second, it conducted a comparative analysis between maternal deaths attributed to COVID-19 and those unrelated to the virus, with the goal of identifying the primary predictors of MM related to COVID-19. Third, the study examined the MMR over time (from 2018 to 2021) and across different governmental administrations. Lastly, it evaluated COVID-19-specific MM and assessed the proportionate MM in 2020 and 2021.

## Materials and methods

### Study design, study setting, and sampling technique

We conducted a record-based cross-sectional analytic study to assess the MM in Egypt during 2020 and 2021, using the MMR as an indicator. The study covered MM brought on by COVID-19 and other pregnancy-related factors. A stratified random sampling technique was employed in this study. Egypt was divided into two strata, Upper and Lower Egypt. In particular, four governorates—two in Lower Egypt (Kafr El-Sheikh and El-Behira) and two in Upper Egypt (Assuit and Fayoum)—were involved in this multicenter study.

### Study population and sample size

A population-based sample of women deaths in the reproductive age group over a 24-month period between 1 January 2020 and 31 December 2021 was obtained using vital registration data. The study included all ever-married women aged 15–49 years who were identified through the national maternal death surveillance system, regardless of parity, provided the death occurred during pregnancy or within 42 days of its termination.

The study excluded all pregnancy-associated deaths resulting from incidental or accidental causes, such as trauma, homicide, or suicide, as these are not classified as maternal deaths according to the ICD-10 definition.

Maternal deaths were classified under two categories. First, deaths directly associated with COVID-19 were included, based on either laboratory confirmation or pulmonary findings suggestive of SARS-CoV-2 infection. Second, deaths resulting from pregnancy-related conditions or those exacerbated by pregnancy were also incorporated.

Using the Power Analysis and Sample Size (PASS) software program (version 20), the minimum required sample size was calculated to be 55,556 live births to estimate the MMR during the COVID-19 period. This calculation assumed an expected MMR of 17 per 100,000 live births, a 95% confidence level (CI), and a margin of error (precision) of 0.0001^7^.

### Data collection

We collected data using the national maternal death monitoring system. Monthly, the national surveillance data is systematically logged and organized in an Excel spreadsheet.

### Study variables

The length of hospital stay, and maternal age were treated as continuous variables. Direct causes of death (e.g., abortion, ectopic pregnancy, antepartum or postpartum hemorrhage, uterine rupture during cesarean section, and hypertensive disorders) and indirect causes of death (e.g., diabetes, thyroid and other endocrine disorders, cardiac conditions, chronic respiratory diseases and renal diseases) were recorded as dichotomous variables (Yes/No).

### Study outcomes

Primary Outcome: The main outcome of this work was to dentify the specific characteristics of maternal deaths occurring in 2020 and 2021 and to calculate the MMR.

$$MMR=\frac{{{\text{Number of maternal deaths}}}}{{{\text{Total number of live births}}}} \times 100,000$$

Secondary outcomes: Secondary outcomes included calculation of cause-specific MM ratios, defined as the number of maternal deaths attributable to a specific cause per 100,000 live births.

$$Cause\,specific\,MMR=\frac{{{\text{Number of maternal deaths due to specific cause}}}}{{{\text{Total number of live births}}}} \times 100,000$$

Additionally, the proportionate MM was estimated by calculating the percentage of maternal deaths out of all deaths among women of reproductive age (usually 15-49 years).

$$Proportionate\,MM(\% )=\frac{{{\text{Number of maternal deaths}}}}{{{\text{Total number of deaths among women aged 15-49 years}}}} \times 100$$

### Statistical analysis

The Statistical Package for Social Sciences (IBM SPSS Statistics for Windows, Version 28.0) was used to analyze data. Descriptive statistics were used to summarize the data. For numerical variables, measures included means with standard deviations as well as medians with ranges. For categorical variables, proportions and percentages were calculated. Pearson’s Chi-square test was used to describe the association between categorical variables. Fisher’s exact test and Montecarlo test were used instead in case of a violation of Pearson’s Chi-square test assumptions. Based on the distribution of the data, independent t-tests or Mann-Whitney tests were used to compare quantitative variables.

The likelihood of maternal death being connected with a COVID-19 diagnosis was estimated using a multivariable logistic regression analysis, which estimates the impact of each independent variable. The logistic regression analysis only included variables that had a significance threshold of *p* < 0.2 or clinical significance.

Before model interpretation, key assumptions of logistic regression were assessed. The outcome variable was binary (Died from COVID-19, Yes/No), and observations were independent. Multicollinearity among predictors was evaluated using the Variance Inflation Factor (VIF), and all values were within acceptable limits (less than 10). Model fit was tested using the Hosmer–Lemeshow goodness-of-fit test, which indicated an adequate fit (*p* > 0.05). The sample size was sufficient relative to the number of predictors, supporting the reliability of the model estimates. The odds ratios (OR) and their corresponding 95%CI for each predictor variable were presented. A p-value < 0.05 was considered statistically significant.

MMR were compared across the years 2018 to 2021 within each governorate using the Chi-square test for trend, to assess any significant temporal patterns. Additionally, comparisons between governorates in the same year were performed using the Chi-square test. COVID-19-specific MM, and proportionate MM were assessed and compared in 2020 and 2021 across the included governorates.

### Ethical approval

The study protocol was reviewed and approved by the Research Ethics Committee of the Ministry of Health and Population (IRB: 00000678). All methods were carried out in accordance with relevant guidelines and regulations. Due to the retrospective nature of the study, Research Ethics Committee of the Ministry of Health and Population waived the need for obtaining informed consent. All data were anonymized and handled with strict confidentiality.

## Results

Table 1 presents the characteristics of the sample, which consists of 541 maternal deaths that occurred between 2020 and 2021. These deaths were recorded among more than 960,000 live births documented in the same period. The distribution of these deaths was the following: 37.7% in Assuit, 28.5% in El-Behira, 22.7% in Fayoum, and 11.1% in Kafr El-Sheikh. Only 11.1% of maternal deaths occurred among individuals aged 20 years or younger, with the overall mean age of the sample being 28.9 ± 6.4 years. In terms of parity, 38.6% of the women had one to two children, 26.8% were nulliparous, and 34.6% had three or more children. A history of previous abortion was reported in 16.1% of the deaths. Regarding antenatal care, the majority (97.6%) of women received routine follow-up either with private doctors, healthcare units, or both. Nearly one-fourth (25.3%) of the women were diagnosed with COVID-19 during pregnancy, and around one-third (34.4%) of them had other comorbidities. Most maternal deaths occurred during the postpartum period (40.8%), followed by the pregnancy period (32.9%) and labour (23.3%). Among the women who died post-delivery, 70.9% had delivered in general hospitals. As for the newborns, 72.3% were born alive and in good condition, whereas 12.4% were alive but required ICU admission. A minority (15.3%) of the newborns died either before or during labour.

**Table 1:** Description of personal, obstetric and mortality characteristics, of maternal deaths recorded from the Egyptian National Maternal Death Surveillance System, 2020–2021 (*N* = 541).

Characteristics		*N*	%
***1. Personal characteristics***			
Residence	Assuit	204	37.7
	El-Behira	154	28.5
	Fayoum	123	22.7
	Kafr El-Sheikh	60	11.1
Age (years)	≤ 20	60	11.1
	> 20 to 30	250	46.2
	> 30	231	42.7
	Mean ± SD	28.9 ± 6.4	
Number of previous deliveries	Nulliparous	145	26.8
	1 to 2	209	38.6
	≥ 3	187	34.6
History of abortion	Yes	87	16.1
	No	454	83.9
***2. Characteristics of last pregnancy***			
Received antenatal care	No	13	2.4
	Private doctor	383	70.8
	Health care units	83	15.3
	Both	62	11.5
Diagnosed with COVID-19	Yes	137	25.3
	No	404	74.7
Presence of other comorbidities with pregnancy	Yes	186	34.4
	No	355	65.6
***3. Mortality characteristics***			
Time of death	During abortion	16	3.0
	During labor	126	23.3
	During pregnancy	178	32.9
	Postpartum	221	40.8
Place of delivery (*N* = 347)	General hospital	246	70.9
	Private center	38	11.0
	Home	34	9.8
	Clinic	29	8.3
Newborn condition (*N* = 347)	Alive (normal)	251	72.3
	Alive admitted to NICU	43	12.4
	Stillbirth	29	8.4
	Intrapartum stillbirth	24	6.9
Surgical intervention	Normal vaginal delivery	152	28.1
	Cesarean section	244	45.1
	Hysterectomy	13	2.4
	D.C after abortion	6	1.1
	Nothing	126	23.3
Year of death	2020	231	42.7
	2021	310	57.3
Place of death	General hospital	348	64.3
	Private hospital	37	6.8
	Private clinic	17	3.1
	Home	105	19.4
	On the road	30	5.6
	Others	4	0.8
Causes of death	**Direct causes**	257	47.5
	Postpartum hemorrhage	79	30.7
	Preeclampsia	66	25.7
	Emboli	40	15.6
	Prepartum hemorrhage	28	10.9
	Abortion (spontaneous or DC)	20	7.8
	Obstructed labor	7	2.7
	Uterine rupture	7	2.7
	Anesthesia related	4	1.6
	Anemia or hyperemesis gravidarum	4	1.6
	Ectopic pregnancy	2	0.8
	**Indirect causes of death**	284	52.5
	Respiratory diseases	129	45.4
	Cardiovascular diseases	100	35.2
	Gastrointestinal diseases	17	6.0
	Neurologic diseases	13	4.6
	Endocrinal disorders	7	2.4
	Cerebral hemorrhage	6	2.1
	Tumors	5	1.8
	Urogenital	4	1.4
	Others	3	1.7
Was there a delayed arrival to the hospital?	No	334	61.7
	Yes	29	5.4
	Not mentioned	178	32.9
Did the deceased mother/relatives refused of hospital referral?	No	332	61.4
	Yes	25	4.6
	Not mentioned	184	34.0
Time to death (in days)	Median (min, max)	2 (0.0, 36.0)	
	Mean ± SD	3.7 ± 4.9	

SD: Standard deviation, min: Minimum, max: Max, DC: Dilation and curettage, NICU: Neonatal intensive care unit.

The percentage of maternal deaths was higher in 2021 compared to 2020 (57.3% vs. 42.7%, respectively). Most deaths (64.3%) occurred in general hospitals. Regarding causes, 47.5% of maternal deaths were attributed to direct obstetric causes [primarily postpartum hemorrhage (30.7%), preeclampsia (25.7%), and embolism (15.6%)]. In contrast, the leading indirect causes of death were respiratory disease (45.4%), followed by cardiac conditions (35.2%). Approximately 5.0% of reported deaths were associated with a delay in reaching hospitals, while a nearly similar proportion involved cases where deseased mothers/relatives declined hospital referral. The median time from hospital admission to death was 2 days, with a range from 0 to 36 day.

According to Table 2, 137 maternal deaths (25.3% of the total sample) were diagnosed with COVID-19. Among these, COVID-19 was identified as the sole cause of death in 103 cases (75.2%). The distribution of COVID-19–related maternal deaths varied significantly across regions, accounting for 48.3% in Kafr El-Sheikh, 22.1% in Assiut, 20.1% in El-Behira, and 26.0% in Fayoum (*p* < 0.001). The proportion of maternal deaths attributed to COVID-19 increased markedly from 15.2% in 2020 to 32.9% in 2021 (*p* < 0.001). Furthermore, approximately one-third (35.1%) of maternal deaths occurring in general hospitals were COVID-19–related, a significantly higher proportion compared to private clinics or home settings (*p* < 0.001). Notably, 88.0% of individuals who refused referral to a hospital and subsequently died were diagnosed with COVID-19, versus only 31.0% among those who accepted referral (*p* < 0.001).

**Table 2:** Comparison of deaths diagnosed with COVID-19 versus Non COVID-19 regarding personal, obstetric and mortality characteristics, recorded from National maternal death surveillance system, 2020–2021.

Variable	Diagnosis of COVID-19		Test of significance (*p*- value)
	Yes (*n* = 137) *n* (%)	No (*n* = 404) *n* (%)	
***Personal characteristics***			
**Residence**			X^2^_3_ = 20.18 *p* < 0.001**
Assuit	45 (22.1)	159 (77.9)	
Kafr El-Sheikh	29 (48.3)	31 (51.7)	
El-Behira	31 (20.1)	123 (79.9)	
Fayoom	32 (26.0)	91 (74.0)	
**Age** (Years)			X^2^_2_ = 4.6 *P* = 0.100
≤ 20	12 (20.0)	48 (80.0)	
> 20 to 30	74 (29.6)	176 (70.4)	
> 30	51 (22.1)	180 (77.9)	
**Median age (min**,** max)**	28 (16, 42)	29 (16, 48)	U = 27398.5 *P* = 0.862
**Mean age ± SD**	28.84 ± 5.69	28.97 ± 6.57	
**Number of past deliveries**			X^2^_2_ = 1.21 *p* = 0.547
Nulliparous	41 (28.3)	104 (71.7)	
1–2	53 (25.4)	156 (74.6)	
≥ 3	43 (23.0)	144 (77.0)	
**History of abortion**			X^2^_1_ = 0.068 *p* = 0.794
Yes	23 (26.4)	64 (73.6)	
No	114 (25.1)	340 (74.9)	
**Characteristics of last pregnancy**			
**Routine follow up**			X^2^_3_ = 2.07 *p* = 0.558
No	5 (38.5)	8 (61.5)	
Private doctor	96 (25.1)	287 (74.9)	
Health care units	23 (27.7)	60 (72.3)	
Both	13 (21.0)	49 (79.0)	
**Presence of comorbidities**			X^2^_1_ = 0.417 *p* = 0.519
No	93 (26.1)	262 (73.9)	
Yes	44 (23.7)	142 (76.3)	
**Mortality characteristics**			
**Time of death**			X^2^_3_ = 26.79 *p* < 0.001**
During abortion	54 (30.3)	124 (69.7)	
During pregnancy	12 (9.5)	114 (90.5)	
During labor	70 (31.7)	151 (68.3)	
Postpartum	1 (6.3)	15(93.8)	
**Newborn condition (*N*** **= 347)**			X^2^_3_ = 2.71 *p* = 0.438
Alive (normal)	59 (23.5)	192 (76.5)	
Alive (admitted to NICU)	9 (32.1)	19 (67.9)	
Intrapartum stillbirth	10 (33.3)	20 (66.7)	
Stillbirth	4 (10.5)	34 (89.5)	
**Year of death**			X^2^_1_ = 22.06 *p* < 0.001**
2020	35 (15.2)	196 (84.8)	
2021	102 (32.9)	208 (67.1)	
**Place of death**			MCP < 0.001**
General hospital	122 (35.1)	226 (64.9)	
Private hospital	5 (13.5)	32 (86.5)	
Clinic	0 (0.0)	17 (100.0)	
Home	6 (5.7)	99 (94.3)	
Road	3 (10.3)	26 (89.7)	
Others	1 (25.0)	3 (75.0)	
**Delayed arrival to hospitals**			X^2^_1_ = 2.03 *p* = 0.15
No	117 (35)	217 (65.0)	
Yes	14(48.3)	15 (51.7)	
**Refuse of hospital referral**			X^2^_2_ = 33.17 *p* < 0.001**
No	103 (31.0)	229 (69.0)	
Yes	22 (88.0)	3 (12.0)	

X^2^_1_ = chi-square test at 1 degree of freedom, U = Mann-Whitney test, MCP = Monte carlo p value, p: p value for comparing between the two studied groups, p value * significant (< 0.5), ** highly significant (< 0.01). NICU: Neonatal intensive care unit, SD: Standard deviation.

As presented in Table 3, a multivariable logistic regression analysis was conducted to examine the impact of various factors on the likelihood of maternal deaths being associated with COVID-19. The model was statistically significant compared to the null model (χ² = 28.69, *p* = 0.001) and showed good fit to the data (*p* for Hosmer-Lemeshow = 0.6). It accounted for approximately 31% of the variation in the probability of COVID-19-related maternal deaths. Maternal age was not significantly associated with COVID-19-related deaths in either the crude [cOR = 1.00; 95% CI: 0.97–1.03] or adjusted models [aOR = 1.03; 95% CI: 0.98–1.08]. In contrast, the year of death was significantly associated, with higher odds in 2021 than in 2020 [cOR = 2.75; 95% CI: 1.79–4.22; aOR = 3.32; 95% CI: 1.90–5.81]. Place of residence in Lower Egypt showed no significant crude association [cOR = 1.26; 95% CI: 0.85–1.87], but became significant after adjustment [aOR = 5.15; 95% CI: 2.61–10.18], suggesting notably higher odds in this region. Refusal of hospital referral was strongly associated with COVID-19 maternal deaths, both in the crude [cOR = 16.3; 95% CI: 4.77–55.7] and adjusted models [aOR = 8.72; 95% CI: 1.73–44.0]. Other factors including number of past deliveries, previous abortions, routine antenatal follow-up, comorbidities, and delays in reaching healthcare facilities, were not significantly associated with COVID-19-related maternal deaths in either univariate or multivariable analyses.

**Table 3:** Predictors of maternal mortality from COVID-19 causes recorded from National maternal death surveillance system, 2020–2021 using a multivariable logistic regression analysis.

Predictor	Unadjusted		Adjusted	
	*p*	OR (95%CI)	*P*	OR (95%CI)
**Age**	0.841	1 (0.97,1.03)	0.217	1.03 (0.98,1.08)
**Year of death** (ref)1	< 0.001**	2.75 (1.79, 4.22)	< 0.001**	3.32 (1.9, 5.81)
**Residence**: (ref)2 Lower Egypt	0.241	1.26 (0.85, 1.87)	< 0.001**	5.15 (2.61,10.18)
**Number of past deliveries**: (ref)_3_	0.548		0.583	
1–2	0.541	0.86 (0.54, 1.39)	0.703	1.14 (0.59, 2.19)
≥ 3	0.273	0.76 (0.46, 1.25)	0.577	0.81 (0.38, 1.71)
**Number of past abortions**	0.981	1 (0.73, 1.36)	0.39	0.84 (0.57, 1.24)
**Routine follow up** (ref)_4_	0.566		0.141	
Private doctor	0.283	0.54 (0.17, 1.68)	0.227	0.44(0.11, 1.68)
Health care units	0.431	0.61 (0.18, 2.07)	0.710	0.76 (0.18, 3.27)
Both	0.187	0.42 (0.12, 1.52)	0.958	0.96 (0.21, 4.39)
**Presence of comorbidities with pregnancy**	0.519	0.87 (0.58, 1.32)	0.8	1.07 (0.63, 1.83)
**Delayed arrival to hospitals**	0.158	1.73 (0.81, 3.71)	0.872	1.1 (0.34, 3.53)
**Refuse of hospital referral**	< 0.001**	16.3 (4.77, 55.7)	0.009*	8.72 (1.73, 44.00)

Model X^2^ = 28.69, df = 5, *p* < 0.001** Nagelkerke R^2^ = 0.31, Hosmer & Lemeshow = 6.39, *p* = 0.6, OR: odds ratio, *p* < 0.001: highly significant** Ref1: 2020, Ref2: upper Egypt, ref3: nulliparous, ref4: no routine follow up. NICU: Neonatal intensive care unit.

**Fig. 1:**
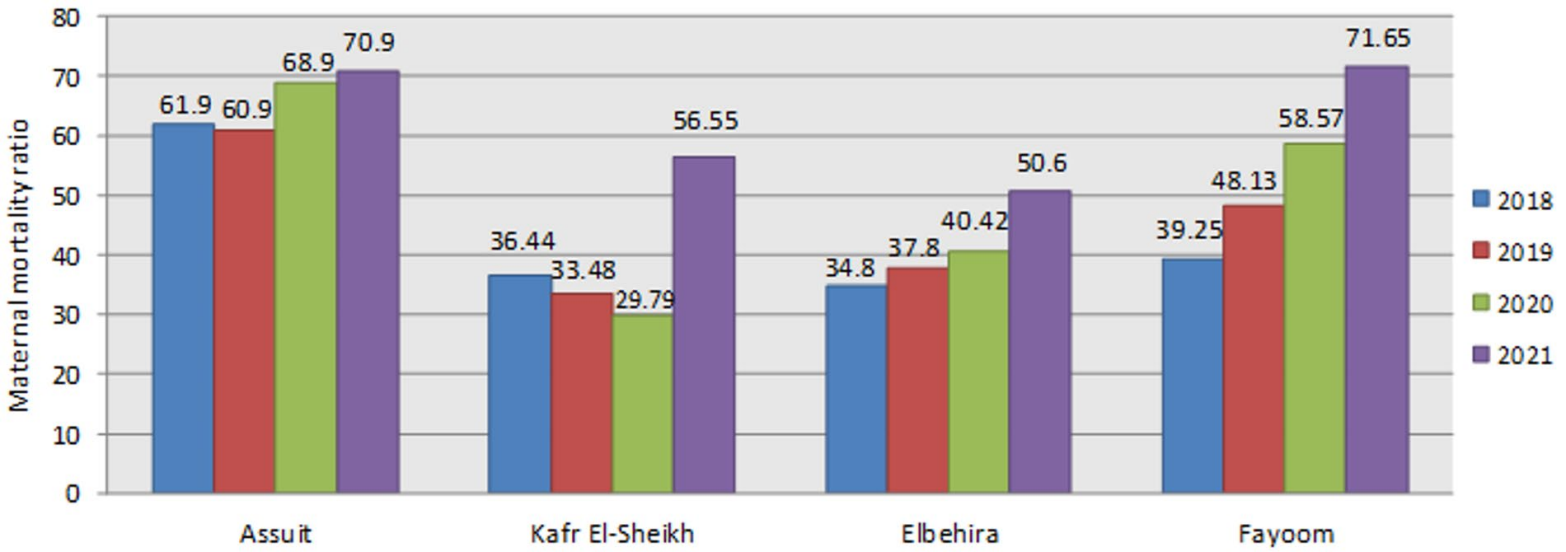
Maternal mortality ratio per 100,000 live births recorded from national maternal death surveillance system, 2020–2021.

As shown in Table 4; Fig. 1, there was an overall rise in MMR from 2018 to 2021. Significant increases were observed in Fayoum (from 39.25 to 71.65; *p* < 0.001) and Kafr El-Sheikh (from 36.44 to 56.6; *p* = 0.032), while El-Behira exhibited a gradual but non-significant rise (*p* = 0.08). Assuit consistently recorded the highest MMR, ranging from 61.9 to 70.9, with no statistically significant change over time (*p* = 0.334).When comparing MMRs across the four governorates within each year, a significant difference was found in all years except 2021 (*p* = 0.15), during which inter-governorate differences were no longer statistically significant. Although the overall MMR increased from 44.1 (pre-COVID-19 period) to 55.9 (post-COVID-19 period), this change was not statistically significant (*p* = 0.236).

**Table 4:** Maternal mortality ratio per 100,000 live births recorded from the Egyptian National Maternal Death Surveillance System, 2018–2021.

	Maternal mortality ratio				Test of significance (P1)
	2018	2019	2020	2021	
Assuit	61.9	60.9	68.9	70.9	0.93 (0.334)
Kafr El-Sheikh	36.44	33.48	29.79	56.55	4.62 (0.032*)
El-Behira	34.80	37.80	40.42	50.6	3.05 (0.081)
Fayoom	39.25	48.13	58.57	71.65	11.11 (< 0.001)*
Test of significance (P2)	11.17 (0.01)*	9.92 (0.019)*	18.81 (< 0.001)**	5.3 (0.15)	
Total	43.0975	45.0775	49.42	62.425	3.74 (0.053)
Total pre& post COVID-19	44.0875		55.9225		1.40 (0.236)

Test of significance: chi-square test *p* < 0.01: significant*, *p* < 0.001: highly significant**.

P1 for comparing MMR within each governorate across years.

P2 for comparing MMR among governments in each year.

**Fig. 2:**
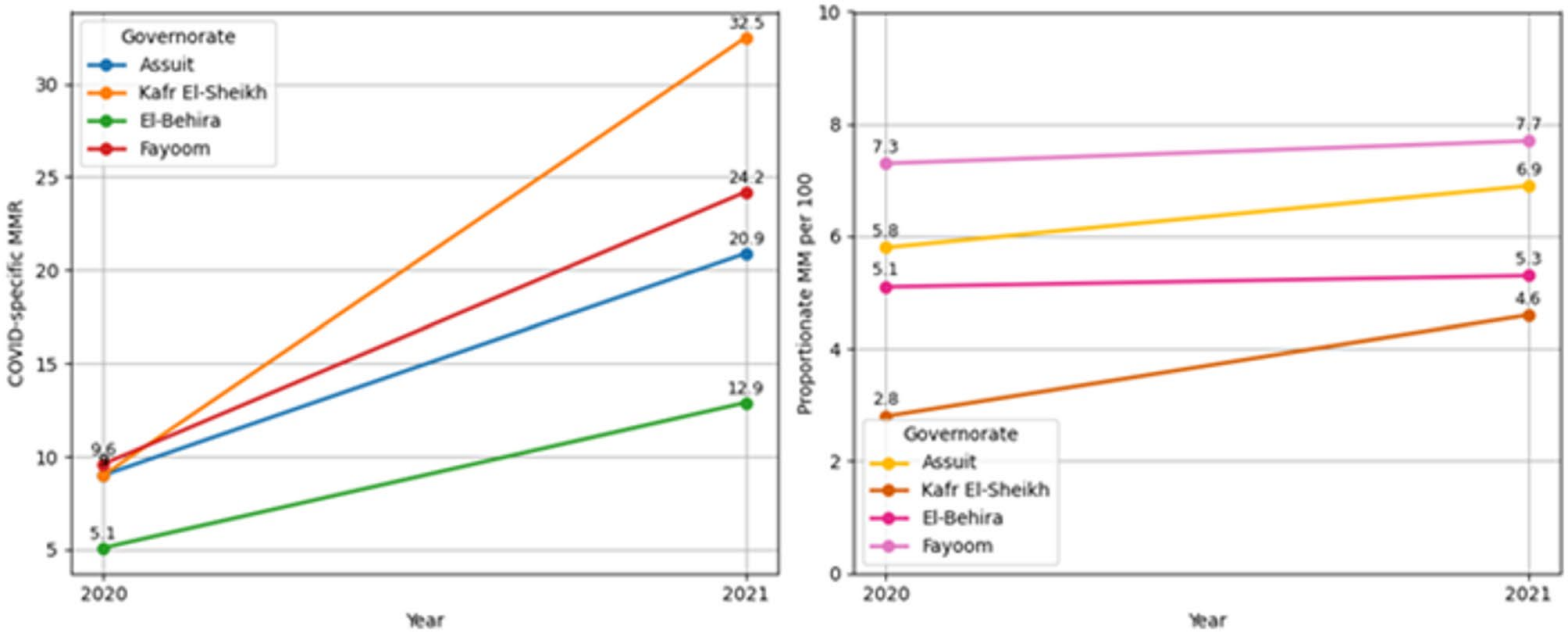
COVID-specific & Proportionate maternal mortality recorded from national maternal death surveillance system, 2020–2021.

COVID-19 specific MM more than doubled nationally between 2020 and 2021 (8.14 to 22.64; *p* = 0.009), with the highest increases in Kafr El-Sheikh (8.94 to 32.52; *p* < 0.001), Fayoum (9.58 to 24.23; *p* = 0.012), and Assuit (8.99 to 20.89; *P* = 0.029). El-Behira’s change was not significant (*p* = 0.063), Fig. 2. When comparing across governorates, no significant differences were found in 2020 (*p* = 0.659). However, in 2021, variation between governorates became statistically significant (*p* = 0.033), with Kafr El-Sheikh recording the highest value as shown in Table 5; Fig. 2.

**Table 5:** COVID-19-Specific and proportionate maternal mortality recorded from the Egyptian National Maternal Death Surveillance System, 2020–2021.

Governorate	COVID-19 specific maternal mortality				Proportionate maternal mortality			
	2020	2021	Both years	Test of significance (P1)	2020	2021	Both years	Test of significance (P1)
Assuit	8.99	20.89	14.94	4.74 (0.029)*	5.77	6.94	6.36	0.11 (0.739)
Kafr El-Sheikh	8.94	32.52	20.73	13.41 (< 0.001)**	2.79	4.62	3.71	0.45 (0.502)
El-Behira	5.05	12.93	8.99	3.45 (0.063)	5.087	5.33	5.21	0.005 (0.943)
Fayoom	9.58	24.23	16.905	6.35 (0.012)*	7.31	7.74	7.53	0.01 (0.921)
Test of significance (P2)	1.6 (0.659)	8.72 (0.033)	4.68 (0.196)		2.02 (0.567)	1.00 (0.801)	1.4 (0.708)	
Total	8.14	22.643		6.83 (0.009)*	5.24	6.16		0.07 (0.792)*

Test of significance: chi-square test *p* < 0.01: significant*, *p* < 0.001: highly significant**.

P1 for comparing COVID-19 specific and proportionate maternal mortality within each governorate across years.

P2 for comparing COVID-19 specific and proportionate maternal mortality among governments in each year.

While proportionate MM increased slightly in 2021 compared to 2020 (5.24 to 6.16 per 100 women, the changes was not statistically significant (*p* = 0.792). Comparisons across governorates in each year revealed no significant inter-governorate differences (*p* = 0.567 in 2020; *p* = 0.801 in 2021). Table 5; Fig. 2.

## Discussion

MM remains a vital indicator of women’s health, reflecting not only medical outcomes but also the accessibility and quality of healthcare systems^21^. The World Health Organization (WHO) has set a target to reduce MMR by two-thirds from its 2010 level by 2030. While many countries have made progress, the global decline has slowed, and in some areas, reversed—particularly during the COVID-19 pandemic^1^. For example, South Sudan reported an alarming MMR of 1,223 per 100,000 live births in 2020, and 95% of global maternal deaths still occur in LMICs, underlining persistent inequities in healthcare systems worldwide^18^. This study aimed to characterize maternal deaths in 2020 and 2021, comparing COVID-19-related deaths with non-COVID-19 deaths. It also identified key determinants of MM due to COVID-19, examined the differences in MMR over time, assessed COVID-19-specific maternal deaths, and the proportionate MM during the study period.

### Maternal mortality pre and post COVID-19

In Egypt, our study identified a significant increase in MM during the pandemic years (2020–2021) compared with 2018 and 2019, aligning with global trends. This increase mirrors findings from Brazil, where 549 maternal deaths occurred in 2020 alone, primarily in the second and third trimesters, and where the MMR rose by 40% during the pandemic^22^. Similarly, in the U.S., MM increased significantly, particularly among racial and ethnic minorities, rural areas, and small cities^23,24^. Interestingly, we found that CS was reported in 45% of MM, a figure that exceeds global averages and highlights a critical area of concern. This finding underscores the urgent need to regulate CS practices in Egypt through educational initiatives and stricter adherence to clinical indications to help mitigate preventable risks^25^. Analysis of antenatal care data revealed that only 15% of women utilized public health units, while over 70% relied on private care. This trend may reflect the public concerns about infection risks in government facilities^26^, which may limit access to essential services.

### COVID-19 associated with maternal deaths

In this study, 25% of maternal deaths were directly attributed to COVID-19. The total number of maternal deaths in 2021 exceeded those in 2020, with COVID-19-related deaths accounting for 32.9% in 2021 compared to 15.2% in 2020. The increase was statistically significant and consistent with a study conducted in Brazil. This study found that pregnant women with COVID-19 faced a significantly higher risk of maternal death, showing a relative risk (RR) of 18.73, with a 95% CI ranging from 11.07 to 31.69^27^. Government-based pattern analysis revealed that Kafr El-Sheikh governorate recorded the highest proportion of COVID-19-related maternal deaths. This geographic variation was statistically significant, underscoring disparities in the regional impact of the pandemic within the same country.

### Predictors of COVID-19-associated MM

Applying multivariable logistic regression, we discovered that MM related to COVID-19 was five times more prevalent in Lower Egypt than in Upper Egypt. Similarly, a national cross-sectional study involving 15,166 participants revealed a COVID-19 infection rate of 46.4% in Lower Egypt compared to 19.7% in Upper Egypt^28^. This discrepancy may be attributed to the variations in disease diagnosis throughout the country. Lower Egypt, being more urbanized and densely populated, likely had better access to diagnostic testing and healthcare facilities, which may have resulted in higher identification and reporting of both COVID-19 cases and related maternal complications. Moreover, environmental factors, population density, and healthcare-seeking behaviours may play a role^29^. A notable predictor of COVID-19 related MM was refusal of hospital referral, which increased the odds of death by over eight times. This behaviour may have stemmed from fear of infection in medical settings and social stigma, or due to the negative impact of the public health and social measures that were strictly implemented during the pandemic^30,31^. In this work, pregnant women in 2021 were more likely to die from COVID-19 than those in 2020. This may be attributed to the emergence of more virulent variants such as Delta, increased strain on the healthcare system, reduced vaccine effectiveness, and reduced adherence to public health precautions^32–34^. Similar trends were reported in the U.S., where the MMR increased from 23.8 per 100,000 live births in 2020 to 32.9 in 2021^35^.

Approximately one-third of the women in our cohort had pre-existing medical conditions. While previous researches, including López-Rodríguez et al.,^36^ has shown that such comorbidities significantly increase the risk of COVID-19-related MM (OR for chronic kidney disease: 4.11; OR for diabetes: 2.53; both *p* < 0.01). Our findings did not replicate this association as comorbidities were not significantly associated with COVID-19-related MM. In our study, increasing maternal age did not emerge as a statistically significant predictor of COVID-19-associated MM. Most of the reported deaths occurred among women aged 20–30, reflecting Egypt’s demographic and cultural context. However, international data suggests higher risk among older mothers. The CDC, for instance, reported that women aged ≥ 40 had a mortality ratio 6.8 times greater than those under 25^35^. Also, Tekin et al.^37^ identified advanced maternal age (above 35 years) as a significant risk factor for COVID-19-related maternal death, particularly during the Delta variant wave. Such variation in findings could be due to differences in population age structure, culture, or the relative scarcity of pregnancies at older ages in Egypt, which may have limited the ability to detect significant associations in this subgroup^38^.

Beyond these clinical factors, maternal outcomes during the COVID-19 pandemic were likely influenced by social and psychological pressures. Studies by Abuhammad et al.^39,40^ in Jordan reported high rates of intimate partner violence during the pandemic being 28.3% among mothers and up to 40% among women generally, especially among those facing unemployment or marital difficulties. These stressors may have worsened maternal health by limiting access to care or increasing stress. While our data did not capture such variables, future research should consider these social factors to better understand risks during health emergencies.

### Maternal mortality ratio

The current analyses revealed a rise in MMRs across several Egyptian governorates between 2018 and 2021, highlighting both temporal and regional disparities. The MMR in Egypt was 43.1 per 100,000 live births in 2018, increased to 49.42 in 2020, and rose more markedly to 62.4 in 2021 which is in line with previous figures reported by the Central Agency for Public Mobilization and Statistics (CAPMAS, 2022) showing an increase of MMR from 43.6 in 2018 to 49 in 2020^41^. However, our figures were slightly higher than those published by the WHO, with numbers ranging from 31.2 to 41.7 per 100,000 live births during COVID-19 era^42^. The difference may be due to international estimates relying on statistical models, using multi-year averages, or using different data sources while our study uses direct annual data from Egypt’s national surveillance system, which may reflect actual maternal deaths and yearly fluctuations.

Notably, Fayoum and Kafr El-Sheikh demonstrated statistically significant increases in maternal deaths, with MMR nearly doubling in Fayoum and showing a sharp incline in Kafr El-Sheikh. This may reflect underlying weaknesses in maternal health services and emergency obstetric care in these regions, consistent with previous literature linking elevated MMR to deficiencies in healthcare infrastructure, particularly in rural or underserved areas^43,44^. While El-Behira exhibited a gradual but non-significant increase, Assuit maintained persistently high MMRs throughout the study period, with no significant change. The sustained elevation in Assuit aligns with earlier national reports identifying Upper Egypt as a hotspot for MM due to delays in accessing timely care and socioeconomic constraints with a reported MMR of 171 in 2013 and 89 in 2014^45,46^. Interestingly, although the inter-governorate comparison showed significant differences in most years, 2021 marked an exception, likely due to a generalized rise in MMRs across all regions. This convergence may have diminished previously apparent disparities, echoing findings from global studies that observed how the COVID-19 pandemic disproportionately strained health systems across the board, levelling out prior regional performance differences^47^.

The overall increase in MMR from 44.1 (pre-COVID) to 55.9 (post-COVID) was not statistically significant. This could be attributed to the variability in pandemic response and healthcare resource allocation between governorates, masking the overall trend when aggregated. Prior research has highlighted that in LMICs, national-level statistics can often obscure sub-national inequalities in maternal health outcomes^48^.

### COVID-19 specific MM

A particularly alarming finding is more than doubling of COVID-19 specific MM between 2020 and 2021 (from 8.14 to 22.64). This sharp escalation was most pronounced in Kafr El-Sheikh, Fayoum, and Assuit, all of which experienced statistically significant increases in MM. These findings are consistent with international studies documenting how COVID-19 heightened maternal risks, especially in settings with limited ICU capacity, testing availability, and timely obstetric interventions^49,50^. By contrast, El-Behira’s increase in COVID-19-related MMR was not statistically significant, possibly reflecting either underreporting or a smaller number of cases ranging from 5.05 to 12.93 per 100,000 live births. Moreover, in 2020, differences in COVID-19-related maternal deaths across governorates were not significant, but these became significant in 2021, suggesting that regional differences in pandemic preparedness and maternal health resilience began to emerge more distinctly as the crisis evolved.

### Proportionate maternal mortality

Finally, the proportionate MM showed a slight, non-significant increase from 2020 to 2021, with no significant inter-governorate differences across years. This apparent stability may be due to the broader impact of COVID-19 on all-cause women’s mortality, not limited to pregnant or postpartum women. In other words, the pandemic likely affected the health and survival of all women, which in turn diluted the visibility of changes specific to MM. Similar observations have been reported globally, where the rise in women’s deaths from both maternal and non-maternal causes during the pandemic masked maternal-specific mortality trends^51^.

### Implications of this research

These findings underscore the urgent need for tailored maternal health strategies that address regional disparities, especially in the context of future health emergencies. Enhanced surveillance, equitable resource distribution, and robust emergency obstetric care are essential to reversing these concerning trends.

### Limitations and strengths

The study’s strengths include its use of the Egyptian national Maternal Death Surveillance System, providing comprehensive MM data across regions and time periods. By covering both Upper and Lower Egypt, it captures regional disparities often missed in single-center studies. The four-year analysis (2018–2021) reveals pre-pandemic and COVID-19 increase in MMR with estimation of COVID-19-specific mortality, and proportionate mortality. Crucially, it identifies key pandemic-era predictors of COVID-19 related maternal death, offering valuable insights for future health policies. However, limitations include its retrospective design, which restricts causal inference, and partial geographic coverage (4 of 27 governorates), despite stratified sampling. Data constraints—such as underreporting, lack of ICU/hospital metrics, and limited postpartum follow-up—may affect accuracy. The limited COVID-19 testing capacity in Egypt, especially in rural or underserved areas, may have led to underreporting or misclassification of COVID-19 related maternal deaths. Similarly, incomplete documentation of comorbidities, socioeconomic variables (e.g., income, violence exposure), or healthcare-related variables, such as ICU availability or hospital capacity further narrow the contextual understanding of the study findings.

## Conclusions

In conclusion, our study highlights a significant rise in MM during the COVID-19 pandemic in Egypt, with marked regional disparities and a clear increase in COVID-19-related maternal deaths between 2020 and 2021. Although the overall increase in MMR during the pandemic compared with the pre-COVID-19 era was not statistically significant, certain governorate—(Fayoum, Kafr El-Sheikh, and Assuit) experienced notable surges, underscoring inequities in healthcare infrastructure and crisis preparedness. COVID-19 accounted for one in four maternal deaths during the study period, and the odds of COVID-related MM were significantly higher in Lower Egypt. Predictors of COVID-19-associated mortality included hospital referral refusal and geographic variation in infection rates. These findings call for urgent, regionally targeted maternal health strategies, improved surveillance systems, and investment in equitable access to obstetric emergency care, especially in underserved areas. Lessons from the pandemic must inform Egypt’s future preparedness plans to mitigate maternal risks during health crises and help meet the MMR reduction target.

## Data Availability

Data is available upon request by emailing the corresponding author.

## Abbreviations

aOR: Adjusted odds ratio
CAPMAS: Central Agency for Public Mobilization and Statistics
CDC: Centers for Disease Control and Prevention
CI: Confidence interval
CKD: Chronic kidney disease
cOR: Crude odds ratio
COVID-19: Coronavirus disease 2019
CS: Cesarean section
D&C: Dilation and curettage
df: Degrees of freedom
ICD-10: International Classification of Diseases, 10th Revision
ICU: Intensive care unit
LMICs: Low- and Middle-Income Countries
min/max: Minimum/Maximum
MM: Maternal mortality
MMR: Maternal mortality ratio
n/N: Number of cases/Sample size
OR: Odds ratio
p: Probability value
Ref: Reference
SARS-CoV-2: Severe acute respiratory syndrome coronavirus 2
SD: Standard deviation
SDGs: Sustainable Development Goals
SPSS: Statistical Package for the Social Sciences
U.S.: United States
VIF: Variance inflation factor
WHO: World Health Organization
χ²: Chi-Square Test
%: Percentage
